# Abnormal Unsaturated Fatty Acid Metabolism in Cystic Fibrosis: Biochemical Mechanisms and Clinical Implications

**DOI:** 10.3390/ijms150916083

**Published:** 2014-09-11

**Authors:** Adam C. Seegmiller

**Affiliations:** Department of Pathology, Microbiology and Immunology, Vanderbilt University School of Medicine, 4918B TVC, 1301 Medical Center Dr., Nashville, TN 37027, USA; E-Mail: adam.seegmiller@vanderbilt.edu; Tel.: +1-615-322-0858; Fax: +1-615-343-9563

**Keywords:** unsaturated fatty acids, cystic fibrosis, fatty acid desaturase, eicosanoids

## Abstract

Cystic fibrosis is an inherited multi-organ disorder caused by mutations in the *CFTR* gene. Patients with this disease exhibit characteristic abnormalities in the levels of unsaturated fatty acids in blood and tissue. Recent studies have uncovered an underlying biochemical mechanism for some of these changes, namely increased expression and activity of fatty acid desaturases. Among other effects, this drives metabolism of linoeate to arachidonate. Increased desaturase expression appears to be linked to cystic fibrosis mutations via stimulation of the AMP-activated protein kinase in the absence of functional CFTR protein. There is evidence that these abnormalities may contribute to disease pathophysiology by increasing production of eicosanoids, such as prostaglandins and leukotrienes, of which arachidonate is a key substrate. Understanding these underlying mechanisms provides key insights that could potentially impact the diagnosis, clinical monitoring, nutrition, and therapy of patients suffering from this deadly disease.

## 1. Introduction

Cystic fibrosis (CF) is one of the most common hereditary disorders of Caucasians, with an incidence of approximately 1 in 3000 live births [1]. It is caused by homozygous or compound heterozygous mutations in the gene encoding the CF transmembrane regulator (CFTR) protein [2,3]. Over 1900 mutations have been described in the *CFTR* gene, the most common of which is deletion of the codon encoding phenylalanine 508 (ΔF508). The CFTR protein is expressed on the apical surface of many epithelial cells and primarily functions as a chloride and bicarbonate transporter, although it has a number of additional transport and regulatory functions [4].

Loss of CFTR function has pathologic consequences in a number of organ systems [5]. These include gastrointestinal obstruction, pancreatic insufficiency and malabsorption, male infertility, and CF-related diabetes and liver disease. However, most morbidity and mortality in CF patients is related to pulmonary disease. In the airways, there are recurring cycles of obstruction, infection, and inflammation, leading to tissue injury, bronchiectasis, and progressive pulmonary decline [6]. This leads to early death in many patients, the average life span extending only to the late 4th decade.

Although considerable progress has been made in understanding the pathogenesis of CF, the connection between loss of CFTR function and the protean clinical and pathologic manifestations of the disease is not completely understood. It is clear that there are factors beyond CFTR that modulate pathology and outcomes, since there is considerable clinical heterogeneity even amongst patients carrying the same mutation [7]. One of these factors is fatty acid metabolism. There are consistent abnormalities in levels of unsaturated fatty acids in patients and models of CF (reviewed in [8,9,10]). This review will describe these changes and discuss what is known about the underlying mechanisms, their connection to *CFTR* mutations and CF pathophysiology, and potential clinical applications of this knowledge in the management of CF patients.

## 2. Unsaturated Fatty Acid Metabolism

Unsaturated fatty acids contain one or more double bonds in the acyl chains. Monounsaturated fatty acids (MUFAs) contain a single double bond, while polyunsaturated fatty acids (PUFAs) carry two or more double bonds. The most common categories of PUFAs are the n-3 or omega-3 and n-6 or omega-6 PUFAs. This nomenclature refers to the position of the most distal double bond, either 3 or 6 carbons from the terminal methyl group of the acyl chain, respectively. These are termed essential fatty acids because their precursors cannot be synthesized by mammalian cells and thus, must be obtained from dietary sources.

**Figure 1:**
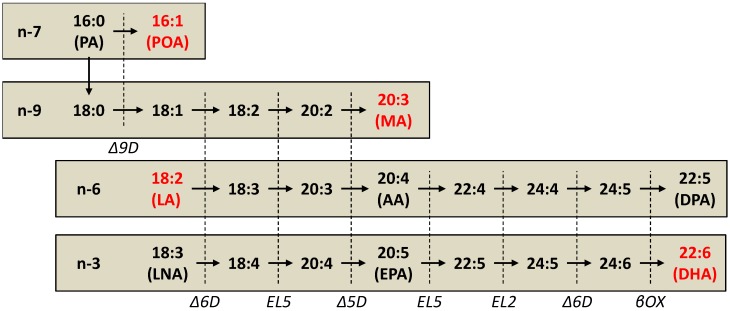
Unsaturated Fatty Acid Metabolism in the n-3, n-6, n-7, and n-9 Pathways. Enzymes are in italics and the vertical dashed line indicates the common reactions that they catalyze. Fatty acids that show consistently abnormal values in blood from cystic fibrosis patients are indicated in red. PA, palmitate; POA, palmitoleate; MA, Mead acid; LA, linoleate; AA, arachidonate; DPA, docosapentaenoate; LNA, alpha-linolenate; EPA, eicosapentaenoate; DHA, docosapentaenoate; Δ9D, Δ9-desaturase; Δ6D, Δ6-desaturase, EL5, elongase 5; Δ5D, Δ5-desaturase; EL2, elongase 2; βOX, β-oxidation.

The primary n-3 and n-6 PUFAs are alpha-linolenate (LNA; 18:3n-3) and linoleate (LA; 18:2n-6). These can be metabolized by mammalian cells to more elongated and desaturated forms via the parallel metabolic pathways shown in Figure 1. These pathways share common elongase and desaturase enzymes, of which Δ6-desaturase is rate-limiting [11]. The activity of these enzymes is regulated primarily at the transcriptional level [12].

Some of these enzymes are also shared by the n-7 and n-9 pathways, which primarily, but not exclusively, metabolize MUFAs (Figure 1). In contrast to PUFA metabolism, these fatty acids are not essential, as they are ultimately derived from palmitate (PA; 16:0), the primary product of *de novo* fatty acid biosynthesis.

## 3. Unsaturated Fatty Acid Levels in Cystic Fibrosis

More than 50 years ago, Kuo *et al.* [13] first identified fatty acid abnormalities in blood and tissues of CF patients. Since this description, there have been at least 18 other reports of altered fatty acid composition in CF, particularly of unsaturated fatty acids, in serum, plasma, erythrocytes, or whole blood [14,15,16,17,18,19,20,21,22,23,24,25,26,27,28,29,30,31]. The most common of these is decreased LA, which is described in all of these studies. Additionally, increases in palmitoleate (POA; 16:1n-7) and Mead acid (MA; 20:3n-9) and decreased docosahexaenoate (DHA; 22:6n-3) have been demonstrated in majority of studies in which they have been measured.

Similar findings have been noted in a smaller number of studies that examined tissues from CF patients. These consistently found decreased LA in adipose tissue, skeletal and cardiac muscle, liver, lung, and nasal epithelium [13,23,27] and decreased DHA in nasal and rectal biopsies [27].

These findings have been confirmed in animal and cell culture systems, which serve as important models for investigating the underlying biochemical mechanisms. Studies in *CFTR*-null [32,33] and *CFTR*-ΔF508 [34] mice show similar abnormalities to those seen in CF patients. These include decreased LA [33,34] that is accompanied by increases in the downstream metabolites dihomo-γ-linolenate (20:3n-6) [33,34] and/or arachidonate (AA;20:4n-6) [32,34] in lung, pancreas, and intestine. Decreased DHA was also observed in one of these studies [32].

In addition, fatty acid metabolism has been examined in two cultured respiratory epithelial cell models of CF. One is 16HBE human bronchial epithelial cells in which CFTR is silenced by stable transfection of a plasmid expressing a *CFTR* antisense oligonucleotide [35]. The other (IB3 cells) is a respiratory epithelial cell line derived from a patient with CF [36]. These models exhibit the same changes as detailed above, including decreased LA and DHA, with increased AA, POA, and MA, when compared with controls [37,38,39,40]. In addition, there are other changes that are not seen in animal or human studies that suggest similar metabolic alterations in the parallel n-3 and n-6 pathways (see Figure 1). For example, both LA and LNA are decreased, both AA and eicosapentaenoate (EPA; 20:5n-3) are increased, and both DHA and docosapentaenoate (DPA; 22:5n-6) are decreased. The observation of these changes in cultured cells, but not whole organisms, is likely due to the fact that these are pure and homogenous populations of cells that are not influenced by the fatty acid composition of blood or other tissues. This makes them particularly valuable for studying underlying biochemical mechanisms.

## 4. Mechanisms of Fatty Acid Abnormalities

Early studies of fatty acid metabolism in CF attributed the observed abnormalities to pancreatic insufficiency and the resultant lipid malabsorption that is typical of CF [20]. In fact, the pattern of fatty acid changes is reminiscent of that seen in patients with essential fatty acid deficiency (EFAD) [41]. Furthermore, abnormal PUFA levels were more common and more severe in patients with pancreatic insufficiency than in those with normal pancreatic function [20,22,23,30].

However, several lines of evidence argue against the malabsorption hypothesis. First, the fatty acid changes persist in CF patients in whom pancreatic insufficiency has been successfully treated with enzyme replacement and aggressive nutritional support [26,29]. Second, in animal models, fatty acid abnormalities are limited to tissues that express high levels of CFTR and are primarily affected by CF, such as lung, pancreas, and intestine [32,34]. Similarly significant changes are not seen in other tissues such as liver, brain, kidney, and heart [32,34]. Finally, the fact that fatty acid abnormalities are present even in cultured cells, in which malabsorption plays no role, suggests that they are related to intrinsic metabolic alterations that are secondary to loss of CFTR function.

An important clue as to the origin of the LA deficiency came in the observation of increased metabolism of AA from LA in tissues [27] and bronchial fluid [42] of CF patients, as well as in animal [32,34] and cell culture models [37,39]. This elevation of the AA/LA ratio suggests increased metabolism of LA to AA. This was confirmed by Njoroge *et al.* [39], who labelled CF cells with ^14^C-LA and demonstrated increased incorporation of the label into cellular AA compared with control cells. This was accompanied by increased expression of both Δ5- and Δ6-desaturase mRNA in CF cells. Similar changes were seen when CFTR activity was blocked by a small molecule inhibitor CFTR_inh_-172.

Further evidence came from DHA supplementation studies. DHA is known to suppress expression of Δ5- and Δ6-desaturases [11,12]. In cultured cells, addition of DHA reduced desaturation expression and normalized LA to AA metabolism, reducing AA levels in CF cells to control-cell levels [43]. Similar results were seen in *CFTR*-null mice, where dietary supplementation with DHA reduced AA to wild-type levels in several tissues [32,34]. Taken together, these results suggest that reduced LA and elevated AA levels are due to increased expression and activity of Δ5- and Δ6-desaturases.

A number of studies have indicated that there is increased release of AA from cell membranes, mediated by cytosolic phospholipase A2 (cPLA2), in cells from CF patients, compared with healthy controls [44,45,46,47,48]. This likely also contributes to the fatty acid changes noted. In particular, the absence of a consistent increase in plasma AA may be a reflection of these changes. Indeed, it is possible that increased LA to AA metabolism is a compensatory response to this change. However, the mechanistic relationship between these two metabolic alterations has not been fully explored.

Similar mechanisms appear to be responsible for the changes in n-7 and n-9 fatty acid metabolism. Levels of POA and MA are increased in many CF patient studies (see above). Similarly, there were increased levels of POA, oleate (18:1n-9), and MA in CF cultured cells compared to controls [40]. Using ^14^C labelling techniques similar to those described above, Thomsen *et al.* [40] demonstrated increased metabolism from PA to POA, OA, and MA in CF cells (see Figure 1). This was accompanied by increased expression of Δ9-desaturase and elongase 6, key enzymes in these pathways.

Although decreased DHA is one of the most common changes seen in CF, its mechanism is the least well understood. It is clear from cell culture studies that metabolism of the upstream precursor EPA to DHA is reduced in CF cells [39]. However, the same study showed no reduction of metabolism of 22:5n-3, the immediate downstream product of EPA, to DHA. There are several potential explanations for this. One possibility is that EPA is preferentially shunted to pathways other than DHA production in CF cells. EPA is a substrate for metabolism by cyclooxygenase to 3-series prostaglandins and by lipoxygenase to 5-series leukotrienes [49], pathways that are both increased in CF cells [39].

Another DHA metabolic pathway is retroconversion of DHA to EPA, reversing typical n-3 metabolism, which occurs through modified β-oxidation in peroxisomes [50,51,52]. This is difficult to assay directly, but the relative activity of this pathway can be estimated by measuring changes in EPA and DHA levels after DHA supplementation [53]. By this technique, there appears to be an approximately 20-fold elevation in DHA to EPA retroconversion in CF *versus* control cells [43]. Thus, one potential cause of reduced DHA levels is disproportionate retroconversion to EPA in CF cells, although this has yet to be validated in other models.

A third hypothesis to explain lower DHA levels in CF involves differences in phospholipid metabolism (reviewed in [10]). The phospholipid phosphatidylcholine (PC) is formed either *de novo*, using dietary choline as a substrate, or by methylation of phosphatidylethanolamine (PE). DHA levels are higher in PC formed via the latter mechanism compared with the former. However, CF cells appear to have a defect in the methyl group metabolism, such that *de novo* synthesis of PC is favored, which could lead to lower DHA levels [54].

## 5. Signalling Pathways Associated with Fatty Acid Abnormalities

The above data provide strong evidence that the alterations in fatty acid levels consistently observed in CF patients and models result from fundamental changes in metabolism that occur in cells lacking CFTR function. However, the connection between CFTR function and fatty acid metabolism is not intuitive. In this context, it is important to recognize that in addition to its role as an ion channel, CFTR also forms complexes with a host of signaling proteins, particularly kinases and phosphatases, that both regulate its function and are regulated by it [55]. Thus, absence of CFTR can disrupt cellular signalling networks with broad functional consequences.

Among the kinases in the CFTR complex is AMP-activated protein kinase (AMPK). AMPK is a heterotrimeric complex of proteins, including a catalytic α subunit and regulatory β and γ subunits. AMPK is a master regulator of cellular metabolism that responds to cellular energy balance by sensing AMP levels and modulating the activity of various metabolic pathways [56,57,58]. In addition, AMPK regulates CFTR activity via phosphorylation [55].

At least two studies using cultured respiratory epithelial cells have demonstrated increased activation of AMPK in CF compared with wild-type respiratory epithelial cells, as measured by increased phosphorylation of AMPKα and AMPK target acetyl CoA carboxylase (ACC) [59,60]. Furthermore, inhibition of AMPK activity normalized Δ5- and Δ6-desaturase expression and activity in CF cells to control cell levels [60]. Exogenous activation of AMPK had the opposite effect, increasing desaturase expression in wild-type cells to CF cell levels. These findings suggest that increased desaturase expression and activity in the absence of CFTR function is mediated by activation of AMPK.

Activation of AMPK is mediated by one of two other kinases, liver kinase B1 (LKB1), which responds to altered cellular AMP levels, and Ca^2+^/calmodulin-dependent protein kinase kinase β (CaMKKβ), which responds to intracellular Ca^2+^ concentration [61,62]. However, while studies have shown no AMP excess in CF cells [59], there is clear evidence of alterations in Ca^2+^ transport and metabolism, leading to increased Ca^2+^ concentration in CF cells [63,64]. Accordingly, inhibition of CaMKKβ, either by a small molecule inhibitor or by Ca^2+^ sequestration, resulted in normalization of desaturase expression and activity in CF cells [60]. These findings suggest that altered Ca^2+^ metabolism and CaMKKβ activity are upstream of AMPK activation in CF.

The mechanism by which AMPK activates desaturase expression is not yet clear. The expression of Δ5- and Δ6-desaturases are known to be activated by the transcription factors peroxisome proliferator-activated receptor alpha (PPARα) [65] and sterol response element-binding protein-1 (SREBP-1) [66,67]. SREBP-1 is unlikely to mediate the effect of AMPK on desaturase expression, as phosphorylation by AMPK inhibits SREBP activity [68]. However, phosphorylation by AMPK activates PGC-1α, a co-activator of PPARα [69,70,71]. Thus, AMPK activation of desaturase expression could be mediated by PPARα. Epigenetic mechanisms could also be considered, as AMPK is also known to mediate transcription by inhibiting histone deacetylases [72,73] or by directly phosphorylating histone H2B [74].

## 6. Fatty Acid Abnormalities and Pathophysiology

As detailed above, there are clear alterations in PUFA metabolism associated with loss of CFTR function that account for the consistent changes in PUFA levels seen in CF. However, the role and significance of these changes in CF disease pathophysiology remain open questions. There are at least three lines of evidence suggesting a connection between metabolism and pathology.

First, PUFA abnormalities correlate with disease severity. Early studies showed that fatty acid alterations, particularly LA and POA levels, were most abnormal in patients with severe pancreatic disease [20,22,23,30]. Strandvik *et al.* [16], showed that patients with *CFTR* mutations associated with more severe disease exhibited lower serum LA and DHA levels than those with other mutations. Furthermore, there appears to be a weak, but statistically significant association between fatty acid levels and pulmonary function [17,18,24,75].

Second, PUFAs and PUFA-derived metabolites play important roles in physiologic pathways of known significance in CF. This was first suggested in two animal models of EFAD that showed CF-like abnormalities in pulmonary immunity and/or inflammation [76,77]. Among the pathways known to be involved in CF are those that metabolize PUFAs to bioactive lipids. These included eicosanoid metabolites of AA, including prostaglandins (PGs), leukotrienes (LTs), and lipoxins (LXs), and docosanoid metabolites of EPA and DHA, the resolvins and protectins.

PG production is increased in CF patients and models [39,78,79,80,81,82] and PG levels correlate with disease severity [78,82,83]. LTs are also increased [39,84,85], especially in acute pulmonary exacerbation [86]. Furthermore, suppression of LT production by corticosteroids appears to be impaired in leukocytes from CF patients [87]. LXs, which suppress inflammation, are decreased in CF [88,89]. There is also increased expression of the metabolic enzymes involved in these pathways in CF patient tissues [90,91] and cultured cells [39,81]. These pathways are particularly important in the regulation of inflammation, which is hyperactive in CF [92], but they likely also play a role in intestinal pathology, which may influence motility [93,94].

Another possibility is that changes in fatty acid metabolism could alter the biophysical properties of epithelial cell membranes, altering the function of membrane proteins. This hypothesis is supported by recent studies indicating that PUFAs can modulate the activity of CFTR and other anion channels [95,96,97,98]. It should be noted that significant abnormalities have also been documented in sphingolipid and cholesterol metabolism in CF [99,100,101]. Although beyond the scope of this review, these changes could also have significant effects on membrane biology in epithelial cells lacking CFTR.

Lastly, reversal of PUFA abnormalities in a mouse model ameliorates CF-related pathology. The *CFTR*^tm1/UNC^ mouse carries a nonsense mutation in exon 10 in the *CFTR* gene, completely eliminating CFTR expression in the homozygous mouse [102]. These mice exhibit pathologic features similar to those of CF patients, especially in the intestines and pancreas [102,103]. As indicated above, these mice show typical PUFA abnormalities, which are reversed by dietary supplementation with DHA [32,34]. In addition, this supplementation ameliorated many of the CF-like pathologic features, including reduction of ileal villus hypertrophy, reversal of pancreatic duct dilation, and suppression of stimulated pulmonary inflammation [32,104]. Particularly intriguing is that this effect in the lungs was accompanied by a significant reduction in PG levels, again suggesting that they may mediate the pathphysiologic effects of PUFA abnormalities in CF [104]. The impact of DHA may have genetic and time components, as the effects on CF-related pathology were not seen in congenic (as opposed to mixed background) mice treated for longer time periods [105].

While not definitive, these studies provide evidence to suggest that abnormal fatty acid metabolism plays a pathologic role in the development of CF. Furthermore, they suggest that correction of the fatty acid defect could potentially impact the clinical course of the disease.

## 7. Clinical Implications

The data described above demonstrate the consistent and integral connection between CF and fatty acid metabolism. There are at least four areas in which this knowledge may be applied to the care of CF patients.

First, fatty acids and their metabolites have potential use in the diagnosis and clinical monitoring of CF. The current standard for diagnosis is screening at birth by a blood test for immunoreactive trypsinogen [IRT], followed by sweat chloride measurement [106]. If positive, the diagnosis is confirmed by genetic testing. However, this approach has challenges, including a high false positive rate of IRT and technical difficulties of sweat chloride [107]. Batal *et al.* [28] demonstrated that the product of serum LA and DHA levels (LA × DHA) is significantly lower in CF patients than in healthy controls. Furthermore, using a blinded approach, they showed that this test could distinguish CF patients from healthy controls with sensitivity that is comparable to sweat chloride without the associated technical challenges. However, specificity was lower, suggesting that a combined approach may be required.

Another area of need in CF clinical care is relevent biomarkers to track the course of disease. Current approaches rely heavily on pulmonary function tests, sputum cultures, and other measurements to diagnose pulmonary exacerbation and monitor response to therapy. However, these measurements suffer from technical challenges and are not adequately sensitive and specific to optimally guide patient care [108]. Thus, there is a significant need to develop clinically and biologically relevant biomarkers that can be measured in blood. There is some preliminary evidence that fatty acid testing could fill that role. As indicated above, fatty acid changes in CF correlate with clinical severity. Furthermore, CF treatment may improve fatty acid status. CF patients successfully treated with lung transplant exhibited absolute fatty acid concentrations similar to healthy controls and significantly different than an age-matched CF population without transplant [109]. Recently, Wojewodka *et al.* [110] demonstrated that PUFAs, in particular AA and DHA, varied during the course of a pulmonary exacerbation, improving with therapy. While these results are suggestive, much more data is required to determine the extent to which fatty acids may serve as relevant clinical biomarkers.

Understanding the mechanisms of fatty acid abnormalities and their connection to CF pathophysiology may also be relevant to dietary therapy. Current recommendations suggest a high-calorie, high-fat diet to maintain body mass in CF patients [111], but they are not specific as to the sources or types of fat, which is an area of increasing concern [112]. Unfortunately, the typical modern western diet has a much higher ratio of n-6 to n-3 PUFAs compared with historic controls [113,114]. Because CF cells have higher LA to AA metabolism, particularly in the setting of high n-6/n-3 ratios [115], the typical CF diet may exacerbate already high levels of AA production, potentially increasing pro-inflammatory PGs and LTs. This concern was confirmed in mouse and cell culture studies showing that LA supplementation increased AA and PG production, leading to increased inflammatory cytokine generation and airway inflammation [116]. Human studies of LA supplementation have given mixed results. Depending on the study, LA-rich dietary supplements have resulted in either no change [21,117,118] or significant increase [119] in blood AA levels. However, tissue AA levels were not measured. LA supplementation appears to have decreased PGF2a production, while increasing PGE2 levels in two small studies [117,120]. However, LA supplementation does appear to improve weight gain in CF infants [118]. While these data suggest that attention should be paid to dietary FA content, and that a lower n-6/n-3 ratio might be beneficial to CF patients, further human studies are required to completely understand the relationship between diet and inflammation.

Finally, these data may suggest a therapeutic approach to CF using PUFAs. As described above, high-dose DHA supplementation reversed some of the CF-related pathology in a mouse model [32]. A number of clinical trials have attempted to assess whether similar supplementation might be effective in CF patients (reviewed in [8,121]). Most of these studies demonstrated improvement in fatty acid levels and decreases in inflammatory markers. Only three studies [122,123,124] showed improvement in clinical performance, such as improvement in pulmonary function tests or decreased exacerbations. However, most of these studies were small (no more than 20 participants) and short (1 year or less). Thus, more extensive clinical trials will be required to definitively assess the potential of this approach to CF therapy.

## 8. Conclusions

Alterations in fatty acid levels are a consistent feature of CF and have been validated both in patients and in multiple models of the disease. Recent studies show that abnormalities in PUFA metabolism underlie these changes and that they may be connected to *CFTR* mutations via the AMPK signalling pathway. Although not definitive, there is compelling evidence suggesting that these abnormalities may play a role in the development of this disease and that understanding the underlying mechanisms may improve understanding of CF pathophysiology. This opens up multiple possibilities for clinical application, from improved diagnosis and clinical monitoring to better nutritional recommendations and therapy.
